# Dual modality feeders: a group of human milk feeders with unique practices and needs

**DOI:** 10.1017/S1368980023002367

**Published:** 2023-12

**Authors:** Ksenia Kholina, Jennifer Brady, Erna Snelgrove-Clarke, Melissa D Rossiter, Kyly C Whitfield

**Affiliations:** 1 Department of Applied Human Nutrition, Mount Saint Vincent University, 166 Bedford Hwy, Halifax, Nova Scotia, B3M 2J6 Canada; 2 Department of Pediatrics, Faculty of Medicine, Dalhousie University, 5850 University Avenue, Halifax, Nova Scotia, Canada; 3 School of Nutrition and Dietetics, Acadia University, 15 University Ave, Wolfville, Nova Scotia, Canada; 4 School of Nursing, Faculty of Health Sciences, Queen’s University, 92 Barrie Street, Kingston, ON, Canada; 5 Department of Applied Human Sciences, University of Prince Edward Island, 550 University Ave, Charlottetown, Prince Edward Island, Canada

**Keywords:** Breast-feeding, human milk, milk expression, lactation support, qualitative research

## Abstract

**Objective:**

Dual modality feeding (DMF) – feeding human milk interchangeably from the breast and from a bottle – comes with unique practical, emotional and relational challenges, as well as support needs. Yet, there is little research that explores the experiences of individuals who use DMF in the Canadian context. The aim of this study is to explore the practices, challenges, reasons and enablers of DMF.

**Design:**

Repeat, semi-structured one-on-one interviews were conducted at 8 weeks and 22 weeks postpartum. Interview transcripts were thematically analysed using a critical feminist lens.

**Setting:**

Nova Scotia, Canada.

**Participants:**

Ten DMF mothers.

**Results:**

DMF practices were influenced by a mix of social and material circumstances, including breast-feeding challenges, the involvement of support persons, finances and access to lactation support. Individuals who predominantly fed at the breast expressed milk strategically to mitigate transitory breast-feeding challenges, for convenience under specific circumstances, and to share feeding responsibilities with other caregivers for personal and practical reasons. Individuals who mainly bottle-fed did so due to long-term breast-feeding challenges or a need to return to employment. Enablers of successful DMF were consistent between the two groups and included practical, personal and relational aspects.

**Conclusions:**

DMF is a unique practice compared to feeding human milk solely from the breast or bottle. Despite the potential growing prevalence of DMF, it is currently understudied and inadequately addressed in existing support programmes in Nova Scotia. Tailored programming and public messaging are needed to support DMF families.

The term breast-feeding in research and public health guidelines is often used synonymously with feeding human milk^(1)^. Likewise, public messaging, programming and other resources about infant feeding typically focus on the promotion of, and best practices for, breast-feeding. However, this focus on breast-feeding overlooks the unique features and needed supports related to expressing human milk (hereafter referred to as expressing or pumping) and/or feeding human milk via other feeding modalities, such as by bottle, spoon, finger or cup^(2)^. The lack of inclusive messaging, programming and resources to support diverse feeding modalities is concerning given that in high-income countries, the use of human milk feeding modalities beyond breast, namely bottle-feeding, is commonplace.

Terminology around the ever-growing complexity of human milk feeding was recently explored in a large Canadian cohort^(3)^. We have introduced the term dual modality feeders (DMF) for those caregivers who feed their own milk both via breast and with a bottle. We propose an inclusive term ‘dual modality feeder’ to refer to individuals of any gender who practice feeding their own human milk at the breast/chest and using alternate modalities (bottle, spoon and cup). By using this terminology, we emphasise that not all individuals who feed their milk to infants identify as mothers. Nevertheless, all participants in this study self-identified as women and as mothers. Therefore, we use this gendered terminology when referring to the participants of this study, according to their self-identification.

The lack of supports for those who express their milk and use feeding modalities other than feeding at the breast may drive caregivers to seek potentially inaccurate or outdated information online or from other sources, rather than from health professionals^(4)^. Moreover, such supports are important enablers for caregivers and their families to navigate an array of aspects, such as physical (i.e. difficulty latching, breast engorgement, mastitis and nipple pain), concerns about under or oversupply^(5–8)^, socio-economic (i.e. need or desire for feeders to return to work^(7,9)^), practical (i.e. involvement of other caregivers, storage, maintaining or increasing milk supply^(10)^) and psycho-social (i.e. desire for bodily autonomy, past trauma and anxiety, avoiding stigma of feeding in public^(8,11)^) factors.

Underlying the lack of supports for DMF is a lack of research that describes the unique experiences, needs and practices of DMF in Canada^(4,12,13)^. Research on DMF has been conducted in the USA but is insufficient to inform policy and best practices in Canada given the distinct parental leave policies, healthcare systems and sociocultural milieu^(14)^. This research helps to fill this gap in the literature and may inform supports that are more responsive to the realities of DMF. More specifically, this research reports on the practices, challenges, reasons and enablers as reported by DMF in Nova Scotia, Canada.

## Methods

### Design

Qualitative descriptive analysis^(15)^ was used to explore the practices and experiences of DMF. This inquiry was guided by breast-feeding self-efficacy theory^(16)^ which has been widely used in breast-feeding research and recently adapted by Fan and colleagues to study reasons for and experiences of human milk expression^(17)^. This theoretical framework was chosen to guide our qualitative exploration of various practical aspects of dual modality feeding. This study was conducted according to the guidelines laid down in the Declaration of Helsinki, and all procedures involving research study participants were approved by the Research Ethics Boards at Mount Saint Vincent University (2020-009) and the University of Prince Edward Island (6008074). Written informed consent was obtained from all participants.

### Setting and relevant context

This study was conducted in Nova Scotia, a province of approximately 1 million people in Atlantic Canada. According to the 2016 Census, 57 % of the Nova Scotian population resided in urban areas, 7 % identified as a visible minority and 17 % were low-income^(18)^. In 2019, the child poverty rate was 24·3 %^(19)^. In 2021, 17·7 % of Nova Scotians were food-insecure, higher than the national average of 15·9 %^(20)^. Key human milk feeding indicators are also relatively lower in Nova Scotia: initiation of 81 % (*v*. 91 % nationally) and exclusive feeding of human milk for 6 months at 30 % (*v*. 35 % nationally)^(21)^.

In Nova Scotia, provincial policy and public health messaging widely promote breast-feeding, but sociocultural norms and structural supports remain inadequate^(22)^. Nova Scotians are still largely uncomfortable with public breast-feeding^(23)^, and appropriate lactation spaces are lacking^(24)^. What is more, public health messages are generally aimed at promoting breast-feeding rather than supporting infant feeding activities and, hence, routinely overlook the unique challenges and required supports of DMF.

### Sample

The participant pool for this study included individuals who practice DMF. Caregivers were eligible to participate if they were 19 years of age or older, lived in Nova Scotia, had a healthy singleton baby who was younger than 8 weeks of age, fed their own human milk at the breast and from a bottle, and planned to exclusively feed human milk up to 6 months. Feeders were not eligible to participate if they had a preterm birth or planned to move out of Nova Scotia within the first 6 months postpartum.

Participants were recruited via convenience sampling with posters displayed in various public spaces and on pertinent social media pages. A total of ten individuals participated in this study. The sample size was decided *a priori* in line with the study design.

### Data collection and analysis

Data were collected between November 2019 and February 2021. Participants completed two semi-structured, one-on-one interviews (at 8 weeks and 22 weeks postpartum) focused on feeding practices and experiences, as well as a demographic questionnaire. Topics explored in the interviews included feeding decisions and routines, formal and informal supports, as well as participants’ views and feelings related to infant feeding and motherhood overall. The second interview was designed to further explore issues identified during the first interview, as well as elicit changes over time. Questions were open-ended, such as ‘Please tell me about your infant feeding routine’ and ‘Please tell me about infant feeding in your family - who is involved in it and why?’ All interviews were conducted by the first author, using the same semi-structured interview guides. Interviews ranged in length from 35 min to 2 h and 38 min. Most of the interviews (*n* 16) were conducted remotely (via Zoom or over the phone) due to the COVID-19 pandemic restrictions; four interviews were completed in-person at participants’ homes. All participants were remunerated with CAD$20 per interview (CAD$40 in total). Interviews were recorded, transcribed verbatim (with names then replaced with pseudonyms), and thematically analysed^(25)^ using MAXQDA 2020^(26)^, with codes defined by the first author and refined with all authors.

## Results

A total of ten individuals participated in the study, all of whom identified as mothers, and used DMF (described in Table 1). Participants were an average of 31·8 (± 5·0) years old, seven were White and eight were multiparous with two to five children. All ten participants were partnered, seven were on paid maternity leave, two had returned to full-time work and one worked part-time at 22 weeks postpartum. The average annual household income was CAD$69 000 (± 26 153). For context, the median annual household income in Nova Scotia in 2021 was CAD$60 200; in Canada – CAD$68 400^(27)^. Most (*n* 8) had breastfed previously, of which five reported a duration of longer than 12 months. In the analysis of the practical aspects of DMF, the four prominent themes included Practices, Challenges, Reasons and Enablers.

**Table 1:** Demographic characteristics of study participants

Characteristic	Participants (*n*)
Ethnicity (mother)	
White	7
First Nations/White	1
Black	1
Latin American	1
Ethnicity (infant)	
White	5
First Nations/White	1
Black/White	1
Latin American/White	2
Other mixed identity	1
Marital status	
Married	8
Common-law	2
Education	
High school diploma	2
College degree	1
Undergraduate degree	6
Graduate degree	1
Parity	
Multiparous	8
Annual household income (CAD)	
<$40 000	1
$40 000–$59 999	3
$60 000–$79 999	3
>$80 000	3
Return to employment at 8 weeks postpartum	
Part-time	1
Full-time	1
Return to employment at 22 weeks postpartum	
Part-time	1
Full-time	2
Predominant feeding modality at 8 weeks postpartum	
Feeding at the breast	8
Bottle-feeding expressed milk	1
Equal breast-feeding and bottle-feeding expressed milk	1
Predominant feeding modality at 22 weeks postpartum	
Feeding at the breast	6
Bottle-feeding expressed milk	1
Equal breast-feeding and bottle-feeding expressed milk	2
Formula-feeding	1
Prior experience of feeding human milk	
<6 months	1
6 months–12 months	2
>12 months	5
Prior experience of formula-feeding	
Yes	4
Mother breastfed as a baby	
Yes	4

### Practices

Participants differed in how they used DMF. Most of the mothers fed their infants predominantly at the breast at both 8 and 22 weeks postpartum (8 and 6 mothers, respectively).

Not surprisingly, mothers performed the primary breast-feeding responsibilities. Nevertheless, mothers also oversaw most of the other feeding-related tasks, such as seeking information, navigating conflicting advice and performing related tasks such as milk storage and bottle sterilisation, including those that enabled the occasional involvement of others who bottle-fed expressed milk. The involvement of other caregivers, such as partners, relatives, older children and friends, was typically described as serving their bonding time and sense of fun, while mothers did the vast majority of daily feeding and associated chores. Bottle-feeding was rarely done by mothers themselves and was instead typically reserved for other caregivers. For instance, Maria (28, Latin American, mother of two) shared:

*The first time I had my child, it was a really big bonding experience – and it still is – to breastfeed and I wanted my husband to have that … [So], we did get him to bottle-feed [our baby] while he was here. I also shared that experience with my son recently … I had him [bottle-feeding] too and it was so cute!*

In addition to infant feeding and associated tasks, virtually all mothers described using human milk for non-feeding purposes. Most commonly, mothers used their milk for ‘milk baths’ or applied it to rashes and sores, as Kimberly (39, White, mother of two) explained:

*I’ve also used it on, like, skin. … If I had a crack or some kind of sore spot on my breast, I would put it on it and leave it. And I’ve also put it on their faces when they’ve had baby acne or, like, little spots that seemed to be a little worse than others or a little cut.*

Multiple mothers in the study also fed human milk to older children mainly due to its perceived immunological properties. Participants described ‘hiding’ human milk in cow’s milk or making human milk popsicles for children to consume without knowledge of its contents.

Moreover, mothers described feeling pressure to save all their milk and find uses for it, even if it was perceived as unsuitable for consumption. Most mothers ended up discarding the milk only when it was perceived to have spoiled. At this point, throwing out the milk was seen as appropriate, emphasising the perceived value of milk and the importance of the amount of effort put into the process of saving it.

Some of the mothers also described creative uses for their milk, such as cooking and baking with their milk, as well as using it for yeast growing and soap making. Additionally, two of the mothers shared that they were planning to get ‘breastmilk jewelry’ made to commemorate their lactation journey.

### Challenges

Mothers experienced a number of challenges with DMF. Supply concerns were prominent among the participants. Although many mothers expressed with a goal of increasing milk supply, our data suggest that supply concerns may actually stem from the ability to visually assess the amount of milk produced when expressing, as explained by Julia (29, second-time mother, First Nations/White):

*I know that … [I] can normally pump five ounces into a bottle, that if I were to go under that, say, three ounces then, I would feel upset. Like, ‘How come that I’m not doing it? How come I’m not producing enough? What’s going on here? Did I drink enough water? Like, what’s going on?’*

Another common challenge associated with DMF is nipple confusion – an infant’s preference for a particular modality of feeding (i.e. breast or bottle) and a difficulty switching between the two. Although the link between the age of initiation of bottle-feeding and the development of nipple confusion has been widely debated, it remained a prominent concern among study participants, as Emma (31, White, mother of two) described:

*In the, like, very, very beginning, … a lot of people are telling you, you know, ‘You don’t want to start bottle-feeding too early or there is nipple confusion’. So, there was a lot of questions with that when she was early-fed. But … it was important to me to start bottle-feeding. So, we started offering it early on. Earlier on than they wanted. [Earlier than] it’s socially acceptable … as far as nipple confusion is concerned but she did great.*

In most cases, mothers reported that nipple confusion resolved with consistent exposure to breast or bottle. The overall lack of information provided to the mothers by healthcare professionals regarding pumping and bottle-feeding was a prominent theme. Tasha (34, White, mother of five) shared the following regarding her experience with her first child:

*I was pumping for him when I was away but I didn’t realise, like, you had to pump on the same frequency as they’d be feeding. Stuff like that I didn’t learn and … the lactation consultant in the hospital didn’t really talk about that with me. So, that was probably the hardest part of our journey – my lack of knowledge about what to do for pumping and ways to … improve it and keep your supply up.*

Similarly, other mothers in the study outlined challenges in accessing evidence-based information and professional advice in this area. Instead, they had to rely on their own experience, online resources and advice from family and friends.

### Reasons

**Fig. 1:**
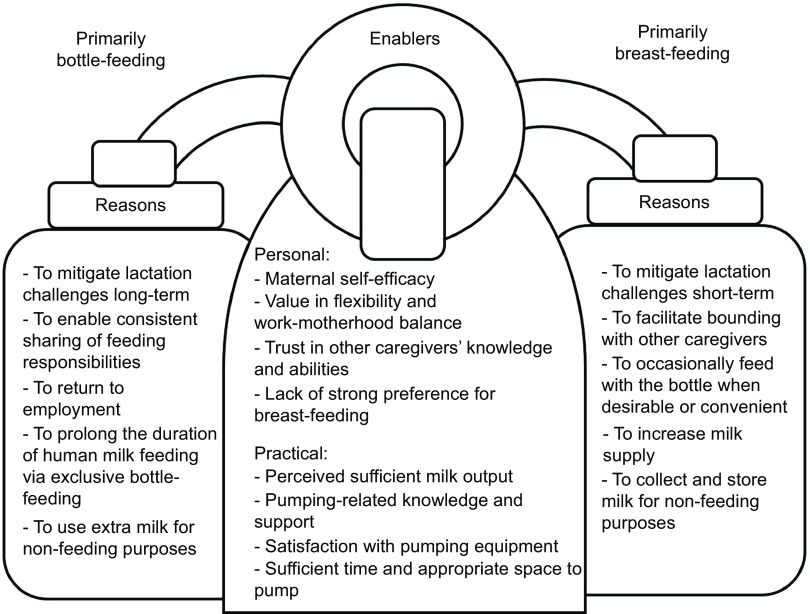
Conceptual framework of reasons and enablers of dual modality feeding in the Canadian context

Mothers shared several reasons for DMF, which were informed by a number of personal and practical enablers, as shown in Fig. 1. The most common reason given for predominant breast-feeding was the greater bonding potential compared to bottle-feeding. For instance, Renée (34, White, first-time mother) explained:

*There’s probably evidence to, like, the euphoric feeling you get when you’re feeding your child. You’re staring into their eyes when you’re breast-feeding, and you know that you’re providing them with the best nutrients possible. And there’s just a bonding experience with breast-feeding *v*. … a bottle is just, like, you need to get them fed … [for] them being full. Whereas [breast-feeding] is nurturing as well, … you’re sharing a moment.*

Another common reason given that shaped choices about feeding modality was convenience. Yet, convenience was contextual and was tied to experience, confidence and setting. Tasha explained:

*With my oldest, it was a little different. I was quite shy about [breast]feeding in public. So, I would actually take a bottle with me to avoid having to feed then. But now, she is my fifth. I’m very comfortable [breastfeeding] and I don’t care who’s around me anymore … The only other time I’ve taken a bottle to bottle-feed since my oldest … was when I was at a wedding, and I had a dress on that wasn’t easy … to open up for him to breastfeed. And then I took a bottle to feed him there because it was just that that time it was going to be easier to bottle-feed.*

Like Tasha, mothers in this study described specific circumstances wherein one feeding modality presented a more convenient option over the other (i.e. being in public, or on car rides). Participants also explained that they preferred to store expressed milk for instances when they were leaving the house, taking a break from breast-feeding or consuming alcohol. Nora (38, White, mother of two) shared:

*So, I am feeding and collecting milk and then when I need to go out, my husband gives him the milk that I’ve collected in a bottle… Or if I just need a break and want to go have a bath and have a glass of wine. And he can give him a bottle!*

Lactation challenges, such as cracked nipples and related latching pain, were another common reason for expression and bottle-feeding. Maria explained:

*I think he latched different[ly] from my first child. He did cause one side of my breasts to bleed. So, I had to pump one side for a while, he drank from the other one while it healed. … It wasn’t as easy as … the first time around.*

Another prominent reason for DMF was return to work, but mothers DMF practices differed with work and family arrangements. For example, Emma indicated a strong preference to return to work and, thus, she and her husband shared responsibilities:

*So, right now I’m self-employed, so days where I have to go to work, I will pump so that he is able to care for our baby during the day and I can go to work … It depends on the day. I might stay home all day, he goes in and does some work or I go in at night … We balance it a lot between the two of us.*

This was different from most families in this study where mothers had a predominant role, as discussed above. Notably, in both of her interviews, Emma shared that she was satisfied with this arrangement. In contrast, Daria, a 27-year-old White immigrant mother of two, also ended up returning to work. Daria explained that she had to do it for immigration reasons as she needed to have worked a certain number of hours to apply for Canadian permanent residency. Since she had a significant preference towards breast-feeding, Daria and her husband came up with a different shared feeding schedule:

*I pump a bottle and he gives that four to five ounces and he also brings the baby to me to my workplace to do an actual feeding … So, I’m at work for about, like, eight to ten hours per day … I breastfeed him before I go to work, once at work and then, my husband gives him one bottle as well.*

However, Daria’s account emphasised the lack of control that she had in regard to this decision as a recent immigrant, making this arrangement emotionally challenging for her:

*I was just, like, missing the baby. I missed being with the baby, seeing him. And I was, like, thinking like, ‘Why on Earth am I here while I should be home with, like, this little human?’ Yeah, and just, overall, I just feel that … for me personally, it was, like, too early to go back to work. But I don’t have much – like, I didn’t have much choice. So, I guess I’m just, you know – I’m trying to, like, stay positive and like, think positive[ly] about it. But … yeah, it’s hard. I miss the baby.*

As can be seen from Emma’s and Daria’s accounts, even when the reason for a particular feeding decision is the same (i.e. bottle-feeding due to return to work), the circumstances around these choices impact the perception of the experience. In addition to return to work, mothers also expressed to prolong the period of exclusive human milk feeding, as explained by Maria:

*My plan is to do it as long as possible honestly. And then, if he does what his brother [did] and bites down on my nipple, then I’m probably just going to pump again … I also need to keep – like, I want to keep my nipple! (laughs). Because we, kind of, do want to plan for another [child] as well. So, yeah. Not right now … but I want to be able to … breastfeed again.*

### Enablers

Enablers of DMF included personal and practical factors. Women connected their primary role in DMF with personal enablers such as maternal confidence and self-efficacy. Hannah (35, White, mother of two) explained:

*I just, kind of, do my own thing and what I think is best … I’ve never sought and nobody has offered really an opinion.*

Trust in caregivers’ ability to periodically care for the infant to allow for mothers’ personal time was another important enabling factor. Nora shared:

*I said that I needed some time off and I defrosted some milk and said [to my husband], ‘The baby can have bottles. I’m locking myself in my room and doing some [video] chats with my friends’. [I] folded laundry while I was in here but yes, it was nice to have a good afternoon … And [I] had a couple drinks … and then, I stopped and was good by the nighttime to be able to do a feed instead of getting up and doing bottles.*

Other practical enablers that shaped choices about feeding modality included satisfaction with and cost of pumping equipment and having sufficient time and appropriate space to express milk. The amount of milk produced was an important visual indicator for the mothers in this study and contributed to the positive perception of expressing, but only when mothers deemed the milk volume to be sufficient. Fernanda (23, Black, first-time mother), who ended up bottle-feeding most of the time due to lactation challenges, shared:

*I pumped off… like, forty millilitres at the time… We bottle-fed him there and he drank it all up. And we were like, ‘Oh my gosh, this is great!’ So, after [that] I saw that he was really eating, and I was obviously producing milk.*

Of note, mothers employed various ways of decreasing the high costs associated with pumping equipment, including renting from a pharmacy, buying second-hand, looking for insurance coverage, or receiving it as a gift.

## Discussion

This study explored the practical aspects of DMF: practices, challenges, reasons and enablers. Importantly, a number of these aspects were different from those previously described in research with breastfeeders and exclusive pumpers, emphasising the clear distinction and support needs between the three groups.

Practices of DMF include feeding at the breast, human milk expression, milk storage, preparation of expressed milk for feeding, bottle-feeding, and cleaning of the equipment and bottles. Although bottle-feeding was commonly presented as an opportunity for other caregivers to get involved, mothers did the majority of daily feeding and chose breast-feeding modality when possible. Involvement of others was occasional, initiated by mothers to allow for a break from feeding, and typically constructed as ‘fun’. This was expected and may be related to the phenomenon of ‘bonding’ through feeding, a prominent theme in women’s encounters around human milk expression^(8)^ and in the context of the contemporary involved fatherhood discourse^(28)^. Other research has shown that feeding as a bonding experience is complicated; some mothers describe feeling pressure to facilitate bonding for other caregivers^(8,29)^, while others experience guilt when their partners bottle-fed the infants, citing perceived infant’s preference for the breast or questioning their partner’s feeding practices^(28)^. This is in line with previous research done with partners that showed that even those who express support and desire to be involved often have little knowledge and unrealistic expectations and, thus, find themselves unprepared for the realities and challenges of human milk feeding^(30)^.

The few notable exceptions to the typical arrangements where mothers performed most of the feeding were due to mothers going back to work before 22 weeks (Emma, Daria) or due to long-standing lactation challenges leading to predominant bottle-feeding (Fernanda). In these cases, although a more equitable feeding schedule was observed, mothers still were mostly responsible for decisions, planning, organising and most associated chores.

Combined with the known importance of partner involvement and support on initiation and duration of human milk feeding^(31,32)^, lack of perceived partner preparedness and the predominance of maternal responsibility across the various DMF arrangements confirms the need for inclusive educational resources and programming. As described above, existing resources and supports do not adequately address other caregivers’ educational needs, leading to possible feelings of inadequacy and lack of involvement^(30)^. For example, *La Leche League*, a well-known peer-support organisation that runs a website with numerous resources on breast-feeding, features a pamphlet entitled *How Partners And Supporters Can Help* ^(33)^. In this document, partner involvement is limited to a solely supportive role, outlining ideas such as bringing snacks to the breast-feeding caregiver, keeping company and doing house chores, without any mention of bottle-feeding.

Similarly, existing public health resources largely focus on breast-feeding and portray bottle-feeding as a last-resort option. For example, *Breastfeeding Basics,* a book produced by the Government of Nova Scotia and given free of charge to the new mothers, does not mention expression or bottle-feeding in the section entitled *How To Feed Your Baby* but does state ‘Be sure your baby’s caregiver understands how to thaw and warm breastmilk safely’ in the section entitled *Feeding Your Baby When You Can’t Be There* ^(34)^. As can be seen from these examples, mothers are positioned as primarily responsible not only for feeding but also for educating others and ensuring their competency, while partner’s role is very limited.

In this study, mothers also experienced common lactation issues, such as nipple pain and poor latch, along with challenges that are unique to DMF, such as nipple confusion. The latter was commonly cited as an anticipated concern or previously experienced challenge. Those who experienced nipple confusion also reported that the issue resolved with time, which aligns with research demonstrating that infants show increased efficiency of feeding with increased familiarity with either mode^(35,36)^. Those who anticipated nipple confusion as a potential problem did so as a result of recommendations from friends or family to delay bottle-feeding for a certain period of time. Support from knowledgeable lactation experts, such as education about the importance of exposure rather than specific age milestones, may redress the challenges faced during DMF.

Another prominent concern identified by participants was related to milk supply. Although supply concerns are common among breastfeeders^(37,38)^, we observed that these were experienced and described in a different way by DMF in this study. As such, mothers were able to visualise and track some (but not all) of their milk output, and thus judge its perceived adequacy based on the measured amount rather than infant’s growth and behaviour. Importantly, recent Canadian research found no significant correlation between actual and perceived insufficient milk supply with the latter being linked with maternal self-efficacy^(39)^. In the future education programming, realistic expectations for milk output, as well as evidence-based strategies to increase milk supply and address common concerns, such as nipple confusion, should be identified and clearly communicated.

Importantly, circumstances around feeding decisions were unique for every family (i.e. bottle-feeding due to preference to return to work *v*. need to do so for immigration reasons), and these impacted mother’s perception of their feeding journey. Thus, healthcare professionals must explore the reasons and circumstances of DMF to provide appropriate care and advice.

Notably, in line with previous literature^(4)^, most of the mothers in this study did not receive any pumping or bottle-feeding-specific resources and had little knowledge prior to their first DMF experience. When seeking information, mothers were faced with inconsistent advice and challenges accessing professional support, leading to disappointment and frustration. This confirms the need for improved public health programming tailored for DMF families, as well as addition of discussion of milk expression in regular prenatal and postpartum care. Additionally, non-commercial information on various equipment options and availability of pump rental programmes needs to be included.

The importance of human milk feeding as a part of the motherhood journey was emphasised by participants when discussing their future plans. Most of the mothers in the study expressed the desire to feed human milk as long as possible, emphasisng the emotional charge of infant feeding and its central place within their overall motherhood experience. In our view, and based on the data, this should be at the centre of support programmes for caregivers, no matter which feeding method they practice.

### Strengths and Limitations

This study adds to a very limited body of knowledge on DMF in high-income countries, identifies unique practices and challenges, and offers new terminology to accurately describe the practices of those who feed both at the breast and using a bottle. In contrast to previous research that often analyses one practice (such as human milk expression or bottle-feeding), this inquiry explored DMF as a combination of intertangled practices in social context. The strengths of this research include its strong theoretical base and methods. Specifically, the longitudinal nature of the study allowed to triangulate findings and note changes during the time between the two interviews.

However, this inquiry is limited to the experiences of Nova Scotian women who practice DMF. Therefore, the results may not be applicable to mothers who either feed only at the breast or who identify as exclusive pumpers, or who mix-feed with human milk substitutes. Additionally, part of the data was collected during COVID-19 pandemic which may have impacted mothers’ experiences, particularly related to feeding in public and availability of in-person supports.

### Conclusions

DMF is distinct from both breast-feeding and exclusive pumping and involves unique practices, challenges, reasons and enablers. Most mothers in the study chose to feed predominantly at the breast and bottle-feeding was typically a backup, mainly to enable bonding with other caregivers or to address short-term lactation issues. Moreover, DMF emphasised the flexibility that bottle-feeding provides for specific circumstances (including returning to work). Regardless of chosen modality, most of the workload and decision-making fell onto mothers who expressed they were inadequately supported to make decisions around feeding due to the lack of easily accessible evidence-based information and professional advice about DMF. The findings of this study emphasise the importance of tailoring future support programmes to DMF families by focusing on mitigating common challenges and fostering maternal self-efficacy.

## References

[1] World Health Organization (2017) World Health Organization Infant Feeding Recommendation. http://www.who.int/nutrition/topics/infantfeeding_recommendation/en/ (accessed June 2021).

[2] Geraghty SR , Sucharew H & Rasmussen KM (2013) Trends in breastfeeding: it is not only at the breast anymore. Matern Child Nutr 9, 180–187. 22625407 10.1111/j.1740-8709.2012.00416.x PMC3448825

[3] Jarman M , Shen Y , Yuan Y et al. (2023) Applying suggested new terminology and definitions for human milk feeding in the Alberta Pregnancy Outcomes and Nutrition (APrON) longitudinal pregnancy cohort. Appl Physiol Nutr Metab 48, 17–26. 36137297 10.1139/apnm-2021-0658

[4] Dietrich Leurer M , McCabe J , Bigalky J et al. (2020) ‘We just kind of had to figure it out’: a qualitative exploration of the information needs of mothers who express human milk. J Hum Lact 36, 273–282. 31710816 10.1177/0890334419883203

[5] Clemons SN & Amir LH (2010) Breastfeeding women’s experience of expressing: a descriptive study. J Hum Lact 26, 258–265. 20689102 10.1177/0890334410371209

[6] Francis J , Mildon A , Stewart S et al. (2020) Vulnerable mothers’ experiences breastfeeding with an enhanced community lactation support program. Matern Child Nutr 16, e12957. 31984642 10.1111/mcn.12957 PMC7296823

[7] Johns HM , Forster DA , Amir LH et al. (2013) Prevalence and outcomes of breast milk expressing in women with healthy term infants: a systematic review. BMC Pregnancy Childbirth 13, 212. 24246046 10.1186/1471-2393-13-212 PMC4225568

[8] Johnson S , Williamson I , Lyttle S et al. (2009) Expressing yourself: a feminist analysis of talk around expressing breast milk. Soc Sci Med 69, 900–907. 19646802 10.1016/j.socscimed.2009.07.001

[9] Felice JP , Geraghty SR , Quaglieri CW et al. (2017) ‘Breastfeeding’ but not at the breast: mothers’ descriptions of providing pumped human milk to their infants via other containers and caregivers. Matern Child Nutr 13, e12425. 28083933 10.1111/mcn.12425 PMC5491362

[10] Felice JP , Geraghty SR , Quaglieri CW et al. (2017) ‘Breastfeeding’ without baby: a longitudinal, qualitative investigation of how mothers perceive, feel about, and practice human milk expression. Matern Child Nutr 13, e12426. 28078789 10.1111/mcn.12426 PMC5491350

[11] Burns E , Schmied V , Sheehan A et al. (2010) A meta-ethnographic synthesis of women’s experience of breastfeeding. Matern Child Nutr 6, 201–219. 20929493 10.1111/j.1740-8709.2009.00209.x PMC6860551

[12] Bigalky J , Dietrich Leurer M , McCabe J et al. (2022) Advice from Canadian mothers who express human milk: an interpretive description qualitative study. Matern Child Health J 26, 342–350. 34609705 10.1007/s10995-021-03237-w

[13] Mildon A , Francis J , Stewart S et al. (2022) Associations between use of expressed human milk at 2 weeks postpartum and human milk feeding practices to 6 months: a prospective cohort study with vulnerable women in Toronto, Canada. BMJ Open 12, e055830. 10.1136/bmjopen-2021-055830 PMC9185489 35676013

[14] Prus SG , Tfaily R & Lin Z (2010) Comparing racial and immigrant health status and health care access in later life in Canada and the United States. Can J Aging Rev Can Vieil 29, 383–395. 10.1017/S0714980810000358 20731891

[15] Sandelowski M (2000) Whatever happened to qualitative description? Res Nurs Health 23, 334–340. 10940958 10.1002/1098-240x(200008)23:4<334::aid-nur9>3.0.co;2-g

[16] Dennis CL & Faux S (1999) Development and psychometric testing of the Breastfeeding Self-Efficacy Scale. Res Nurs Health 22, 399–409. 10520192 10.1002/(sici)1098-240x(199910)22:5<399::aid-nur6>3.0.co;2-4

[17] Fan HSL , Fong DYT , Lok KYW et al. (2023) A qualitative exploration of the reasons for expressed human milk feeding informed by the Breastfeeding Self-Efficacy Theory. J Hum Lact 39, 146–156. 35414281 10.1177/08903344221084629

[18] Statistics Canada (2017) Census Profile, 2016 Census - Nova Scotia and Canada. https://www12.statcan.gc.ca/census-recensement/2016/dp-pd/prof/details/page.cfm?Lang=E&Geo1=PR&Code1=12&Geo2=PR&Code2=01&SearchText=Canada&SearchType=Begins&SearchPR=01&B1=All&type=0 (accessed May 2022).

[19] Frank L , Fisher L & Saulnier C (2021) 2021 Report Card on Child and Family Poverty in Nova Scotia. https://policyalternatives.ca/publications/reports/2021-report-card-child-and-family-poverty-nova-scotia (accessed August 2022).

[20] Tarasuk V , Li T & Fafard St-Germain AA (2022) Household Food Insecurity in Canada, 2021. https://proof.utoronto.ca/resource/household-food-insecurity-in-canada-2021/ (accessed August 2022).

[21] Chan K , Labonté JM , Francis J et al. (2023) Breastfeeding in Canada: predictors of initiation, exclusivity, and continuation from the 2017–2018 Canadian Community Health Survey. Appl Physiol Nutr Metab 48, 256–269. 36596236 10.1139/apnm-2022-0333

[22] Kirk SFL , Sim SM , Hemmens E et al. (2012) Lessons learned from the implementation of a provincial breastfeeding policy in Nova Scotia, Canada and the implications for childhood obesity prevention. Int J Environ Res Public Health 9, 1308–1318. 22690194 10.3390/ijerph9041308 PMC3366612

[23] Chan K & Whitfield KC (2022) ‘Too old’ and ‘too cold’: discomfort towards photographs of breastfeeding beyond infancy and public breastfeeding in Nova Scotia, Canada. J Hum Lact 38, 353–363. 34549657 10.1177/08903344211046191 PMC9016677

[24] West JM , Power J , Hayward K et al. (2017) An exploratory thematic analysis of the breastfeeding experience of students at a Canadian University. J Hum Lact 33, 205–213. 28135477 10.1177/0890334416679621

[25] Attride-Stirling J (2001) Thematic networks: an analytic tool for qualitative research. Qual Res 1, 385–405.

[26] VERBI Software (2021) MAXQDA 2022. https://www.maxqda.com/ (accessed June 2021).

[27] Statistics Canada (2023) Median After-Tax Income, Canada and the Provinces, 2017 to 2021. https://www150.statcan.gc.ca/n1/daily-quotidien/230502/t002a-eng.htm (accessed July 2023).

[28] Ryan K , Team V & Alexander J (2013) Expressionists of the twenty-first century: the commodification and commercialization of expressed breast milk. Med Anthropol 32, 467–486. 23944247 10.1080/01459740.2013.768620

[29] Leeming D , Williamson I , Lyttle S et al. (2013) Socially sensitive lactation: exploring the social context of breastfeeding. Psychol Health 28, 450–468. 23126658 10.1080/08870446.2012.737465

[30] Sihota H , Oliffe J , Kelly MT et al. (2019) Fathers’ experiences and perspectives of breastfeeding: a scoping review. Am J Mens Health 13, 155798831985161. 10.1177/1557988319851616 PMC6537273 31092114

[31] Al Namir H , Brady A & Gallagher L (2017) Fathers and breastfeeding: attitudes, involvement and support. Br J Midwifery 25, 426–440.

[32] Wang S , Guendelman S , Harley K et al. (2018) When fathers are perceived to share in the maternal decision to breastfeed: outcomes from the Infant Feeding Practices Study II. Matern Child Health J 22, 1676–1684. 29961230 10.1007/s10995-018-2566-2

[33] La Leche League (2022) How Partners and Supporters Can Help. https://www.lllc.ca/sites/default/files/How%20Partners%20and%20Supporters%20Can%20Help-11.pdf (accessed July 2023).

[34] Province of Nova Scotia (2015) Breastfeeding Basics. http://novascotia.ca/dhw/healthy-communities/documents/Breastfeeding-Basics.pdf (accessed July 2023).

[35] Ventura A , Hupp M & Lavond J (2021) Mother–infant interactions and infant intake during breastfeeding *v.* bottle-feeding expressed breast milk. Matern Child Nutr 17, e13185. 33939269 10.1111/mcn.13185 PMC8476436

[36] Taki M , Mizuno K , Murase M et al. (2010) Maturational changes in the feeding behaviour of infants - a comparison between breast-feeding and bottle-feeding. Acta Paediatr 99, 61–67. 19839957 10.1111/j.1651-2227.2009.01498.x

[37] Gatti L (2008) Maternal perceptions of insufficient milk supply in breastfeeding. J Nurs Scholarsh 40, 355–363. 19094151 10.1111/j.1547-5069.2008.00234.x PMC4508856

[38] Wood NK & Sanders EA (2018) Mothers with perceived insufficient milk: preliminary evidence of home interventions to boost mother–infant interactions. West J Nurs Res 40, 184–202. 10.1177/0193945916687552 28322655

[39] Galipeau R , Dumas L & Lepage M (2017) Perception of not having enough milk and actual milk production of first-time breastfeeding mothers: is there a difference? Breastfeed Med 12, 210–217. 28326807 10.1089/bfm.2016.0183

